# Metabolic Effects of Hypoxia in Colorectal Cancer by ^**13**^C NMR Isotopomer Analysis

**DOI:** 10.1155/2014/759791

**Published:** 2014-07-01

**Authors:** Ana M. Abrantes, Ludgero C. Tavares, Salomé Pires, João Casalta-Lopes, Cândida Mendes, Marta Simões, Manuela M. Grazina, Rui A. Carvalho, Maria Filomena Botelho

**Affiliations:** ^1^Biophysics Unit, IBILI, Faculty of Medicine, University of Coimbra, 3000-548 Coimbra, Portugal; ^2^Centre of Investigation on Environment, Genetics and Oncobiology (CIMAGO), Faculty of Medicine, University of Coimbra, 3001-301 Coimbra, Portugal; ^3^Life Sciences Department, Faculty of Sciences and Technology, University of Coimbra, 3000-456 Coimbra, Portugal; ^4^Faculty of Sciences and Technology, University of Coimbra, 3000-456 Coimbra, Portugal; ^5^Radiotherapy Department, CHUC, 3000-075 Coimbra, Portugal; ^6^Laboratory of Biochemical Genetics (CNC/UC), Faculty of Medicine, University of Coimbra, 3000-548 Coimbra, Portugal; ^7^Center for Neuroscience and Cell Biology (CNC), 3004-517 Coimbra, Portugal

## Abstract

^13^C NMR isotopomer analysis was used to characterize intermediary metabolism in three colorectal cancer cell lines (WiDr, LS1034, and C2BBe1) and determine the “metabolic remodeling” that occurs under hypoxia. Under normoxia, the three colorectal cancer cell lines present high rates of lactate production and can be seen as “Warburg” like cancer cells independently of substrate availability, since such profile was dominant at both high and low glucose media contents. The LS1034 was the less glycolytic of the three cell lines and was the most affected by the event of hypoxia, raising abruptly glucose consumption and lactate production. The other two colorectal cell lines, WiDr and C2BBe1, adapted better to hypoxia and were able to maintain their oxidative fluxes even at the very low levels of oxygen. These differential metabolic behaviors of the three colorectal cell lines show how important an adequate knowledge of the “metabolic remodeling” that follows a given cancer treatment is towards the correct (re)design of therapeutic strategies against cancer.

## 1. Introduction 

Cancer cell alters its metabolism in response to a challenging environment by promoting cell growth and proliferation, diverging significantly from normal tissues. According to Otto Warburg, cancer cells maintain high aerobic glycolytic rates and produce high levels of lactate and pyruvate [1–3] to sustain cell proliferation and its high energy demands. When the pO_2_ is normal, the oxidative phosphorylation process occurs and pyruvate is directed towards the Krebs cycle. Thus, metabolism in “Warburg” like tumor cells could at first be seen as “wasteful” when compared to normal cells, or we could say that tumor cells use a disproportionate nutrient exchange with its environment. This metabolic profile is in fact prevalent in many cancer cells and grants them advantage over normal cells by allowing them to proliferate at much higher rates. By avoiding oxidative phosphorylation even when oxygen is plentiful [4] and adopting aerobic glycolysis, carbon skeletons build up considerably in their cytosol and biosynthetic pathways are efficiently activated. Several studies have demonstrated an increase in the contents of several glycolytic enzymes such as 6-phosphofructo-2-kinase/fructose 2,6-biphosphatases. The regulation of glycolysis by these enzymes allows the alterations in glycolytic fluxes required to fulfill cancer cells bioenergetics and biosynthetic demands. The glycolytic pathway is in fact becoming an increasing target in cancer therapy both by itself and in combination with other therapies such as immunotherapy. This advance helps overcoming drug resistance issues and improves the efficacy of current anticancer agents [3, 5–7].

Besides that, and having into account tumor microenvironment, due to the temporal and spatial heterogeneity of oxygenation that occurs in solid tumors, the adaptation to the variability of its microenvironment is critical. Oxygen supply is impaired in many tumors because there is imbalance between tissue growth and the development of new vasculature. In solid tumors hypoxia is thus a common characteristic/microenvironment of tumoral cells, becoming a key factor for tumor progression and resistance to anticancer therapy [8]. This decrease in oxygen pO_2_ influences compensatory physiological events involving adaptations at all levels in order to maintain homeostasis between cells energetic requirements and supplies [8]. Thus, it appears that aerobic glycolysis is an adaptive mechanism that involves several metabolic pathways coordinates, which maintain the morphological characteristics of tumor cells, including the ability to survive hypoxic conditions, the capacity of metastasis, and evasion of death by apoptosis [5–8].

Solid tumors have also heterogeneous populations of cells due, in part, to a limited blood supply that provides reduced levels of oxygen and prompts for acidic conditions and avidity of glucose [9]. These changes in the tumor microenvironment may represent physiological signals that activate cell survival or death by apoptosis, affecting the balance between growth and tumor suppression. The mechanisms by which tumor cells adapt or die in the presence of low levels of oxygen are not well studied and understood. However, it is known that the expression of several transcription factors as well as the modification of metabolic pathways interfere with the response to the lack of oxygen and nutrients by tumor cells [10].

The aim of this study is to characterize the metabolic profile, namely, glycolysis and Krebs cycle fluxes, of three colorectal cancer cell lines using carbon-13 (^13^C) tracers and nuclear magnetic resonance (NMR) spectroscopy. With this approach central metabolic pathways will be evaluated and major metabolic changes resulting from hypoxia and glucose availability will be determined towards depicting the possible involvement of metabolic mechanisms [11] in some processes of chemotherapeutic resistance in colon cancer [12].

## 2. Materials and Methods 

### 2.1. Cell Lines

Colorectal cancer cell lines (WiDr, LS1034, and C2BBe1) purchased from American Type Culture Collection (ATCC, Rockville, MD, USA) were used to perform* in vitro* studies. The three cell lines are from different colon localizations: WiDr from rectosigmoid location, C2BBe1 clone of the cell line Caco-2 (ATCC) from descending colon, and LS1034 resistant to chemotherapy concerning P-glycoprotein overexpression from ascending colon [9–13]. WiDr and C2BBe1 cell lines were maintained in Dulbecco's Modified Eagle's cell culture medium (Sigma) and LS1034 cell line was maintained in Roswell Park Memorial Institute (RPMI) medium. All cell lines were cultured in high (25 mM) and low (5 mM) glucose concentrations, supplemented with 10% fetal calf serum (Gibco) in 5% CO_2_ atmosphere, at 37°C. To perform hypoxia studies, cells were incubated at 37°C in 93% N_2_, 2% O_2_, and 5% CO_2_ using a controlled-environment cabinet (PlasLabs, USA). Each of the media was acquired without glucose in order to be supplemented with [U-^13^C]glucose for performing ^13^C NMR isotopomer analysis.

### 2.2. Cell Perchloric Acid Extracts

Perchloric acid extracts were obtained using published methods [14, 15]. Briefly, to the cell pellet is added the equivalent (v/w) of 2 mL ice-cold perchloric acid 7.2%/gram wet weight of cells. The resulting supernatant is subsequently neutralized using ice-cold KOH solutions before lyophilization. This procedure allows precipitation of KClO_4_ and a considerable reduction of solution ionic strength towards the acquisition of high quality NMR spectra. For kinetic analysis of lactate production, aliquots of cell culture media (160 *μ*L) were drawn at specific time intervals (0, 4, and 8 hours). To each of these aliquots 40 *μ*L of an internal standard consisting of sodium fumarate (10 mM) in phosphate buffer (100 mM) was added for absolute quantification purposes.

### 2.3. NMR Analyses of Intermediary Metabolism

#### 2.3.1. Glycolytic Fluxes by Analysis of Cell Culture Media

**Figure 1:**
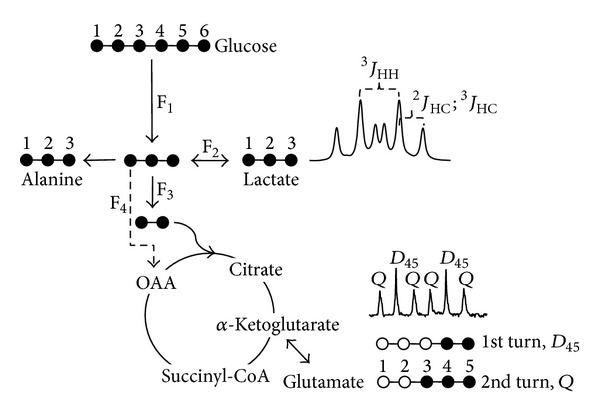
[U-^13^C]glucose as a carbon tracer for monitoring glycolysis and Krebs cycle. Through glycolysis and lactic fermentation [U-^13^C]lactate is produced which is distinguishable (multiplets in each ^13^C satellite due to ^2^ *J*_HC_, ^3^ *J*_HC_, and ^3^ *J*_HH_—duplet of triplets since ^2^ *J*_HC_ and ^3^ *J*_HC_ have similar magnitude) from tissue lactate or lactate originating from unenriched carbon sources. [1,2-^13^C_2_]acetil-CoA originating from [U-^13^C]pyruvate leads to incorporation of ^13^C atoms in Krebs cycle intermediates and other metabolites that exchange with those (e.g., glutamate). It is possible to follow Krebs cycle kinetics by monitoring the ^13^C multiplet due to carbon 4 of glutamate. The duplet component (*D*_45_) results from the simple entrance of [1,2-^13^C_2_]acetil-CoA but the quartet requires further cycling and combination of [1,2-^13^C_2_]acetil-CoA with previously enriched oxaloacetate (OAA). A simple ratio of* Q*/*D*_45_ is proportional to Krebs cycle flux and denotes higher rates of oxidative metabolism in tissues as opposed to glycolysis. Metabolic fluxes F_1–4_ denote the major pathways for [U-^13^C]glucose use.

Proton (^1^H) nuclear magnetic resonance (NMR) spectra of cell culture media were acquired on a 600 MHz Varian NMR spectrometer using a 3 mm indirect detection probe. Typical acquisition parameters included a 3 s acquisition time, a radiofrequency pulse of 45°, and an interpulse delay of 10 seconds to ensure full relaxation of all nuclei in the sample. Due to direct (^1^ *J*_HC_) and long range (^2^ *J*_HC_  and^3^ *J*_HC_) heteronuclear scalar coupling it is possible to distinguish the resonances of [U-^13^C]lactate from those due to nonenriched [U-^12^C]lactate (Figure 1). [U-^13^C]lactate originates from [U-^13^C]glucose as a result of glycolysis followed by reduction of pyruvate by lactate dehydrogenase (LDH). Levels of [U-^13^C]lactate are thus used as an indirect measure of glycolytic activity.

#### 2.3.2. Krebs Cycle Kinetics by ^1^H and ^13^C NMR Analysis of Cell Extracts

^1^H- and ^13^C-NMR spectra of cell extracts were acquired in a 600 MHz Varian NMR spectrometer using a 3 mm indirect detection and a 3 mm broadband NMR probe, respectively. ^1^H-NMR spectra of cell extracts allow the monitoring of the levels of intracellular metabolic intermediates while ^13^C-NMR spectra allows tackling the dynamics of ^13^C incorporation in metabolic intermediates thus warranting a dynamic vision of cell metabolism. Rates of ^13^C incorporation in glycolytic and Krebs cycle intermediates are used as a measure of metabolic coupling between these two major metabolic pathways and provide valuable clues about the “metabolic remodeling” associated with the tumorigenic transformation and treatment. ^13^C isotopomers of intracellular glutamate provide a window for monitoring Krebs cycle kinetics (Figure 1) since the appearance of multi-^13^C-labeled species clearly indicate cycle turnover [14, 16, 17] and oxidative efficiency.

### 2.4. Citrate Synthase Enzyme Activity

The citrate synthase enzyme (CS) is used as a marker enzyme of mitochondrial preparations due to its stability and its regulatory role in the Krebs cycle. Its activity can provide an estimate of the number of mitochondria in a cell suspension and can be used in order to standardize the results of the activities of the enzymes of the mitochondrial respiratory chain [15, 18]. The evaluation of the activity of CS was performed by spectrophotometry at a *λ* of 412 nm. This assay consists of the condensation reaction of acetyl-CoA (0.2 mM) with oxaloacetate (8 mM), catalyzed by CS. This reaction results in coenzyme A (CoA) release that reacts with 5,5′-dithiobis(-nitrobenzoic acid) (DNTB) added to the medium at a concentration of 2 mM, thus enabling the reading at 412 nm. To allow the access of substrates to the enzyme Triton X-100 was used at 0.1%.

### 2.5. Complex IV Activity

To measure complex IV activity, 50 *μ*g of protein was added to 1 mL of buffer composed by 10 mM KH_2_PO_4_, 300 mM sucrose, and 5 mg/mL BSA with pH 6.5, which was in a cuvette. The analysis started with the completion of a baseline tracing at *λ* 550 nm and then 10 *μ*L was added of detergent n-dodecyl-*β*-D-maltoside, which promotes the formation of pores in the outer membrane of mitochondria that allow the entry of reduced cytochrome c, resulting in a concentration of 125 mM. This detergent transfers its electrons to cytochrome c from the mitochondrial respiratory chain located in the inner mitochondrial membrane, without the inner membrane commitment. Upon addition of reduced cytochrome c solution to make up the concentration of 10 mM, it becomes possible to record changes in absorbance at 550 nm. At the end, 2 *μ*L of a solution of potassium cyanide (KCN) 80 mM was added to inhibit complex IV. Measuring the ratio of complex IV activity in the presence and absence of detergent allows the analysis of mitochondrial fraction quality.

### 2.6. Statistical Analysis

Statistical analysis of the different results was performed using the IBM SPSS software v. 20.0 (IBM Corporation, Armonk, New York, USA). Doubling time results were analyzed by nonparametric Kruskal-Wallis test, with multiple comparisons using Bonferroni correction. NMR results were analysed using parametric Student* t*-test. Complex IV activity results were analyzed using nonparametric Kruskal-Wallis test. A significance of 5% was considered for all comparisons.

## 3. Results and Discussion 

### 3.1. Rates of Glycolysis

**Figure 2:**
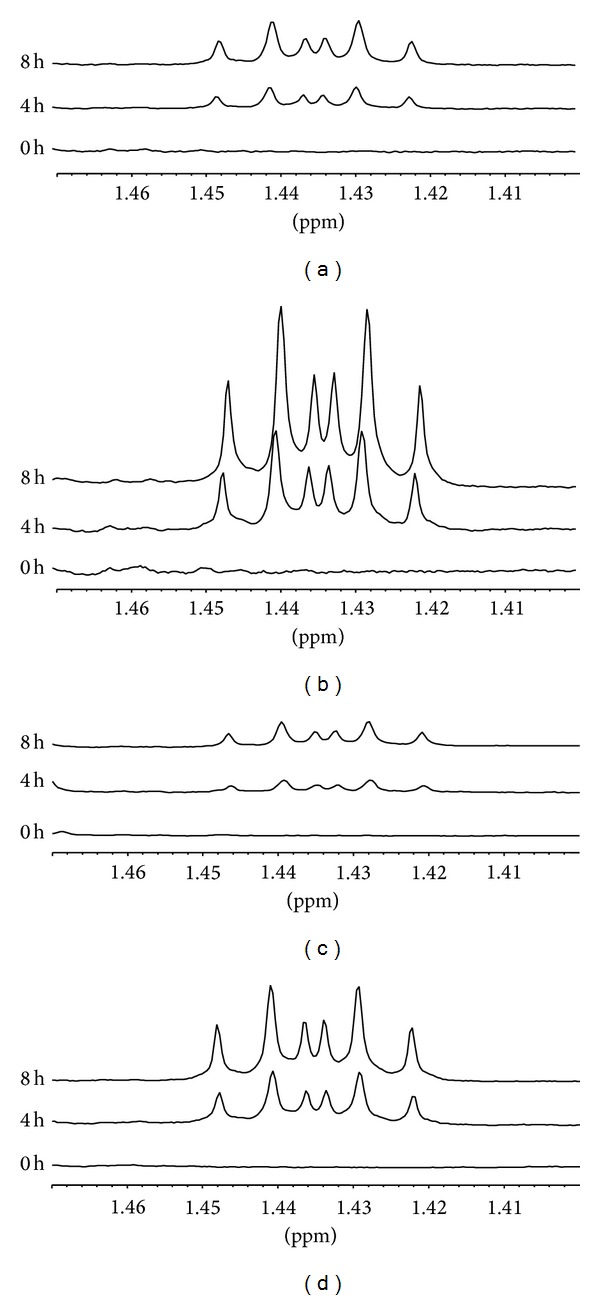
Expansions from ^1^H-NMR spectra of culture media of LS1034 cells, showing the region of one of the satellites due to [U-^13^C]lactate at 0, 4, and 8 hours of incubation in culture media: (a) normoxia/high glucose; (b) hypoxia/high glucose; (c) normoxia/low glucose; (d) hypoxia/low glucose.

**Figure 3:**
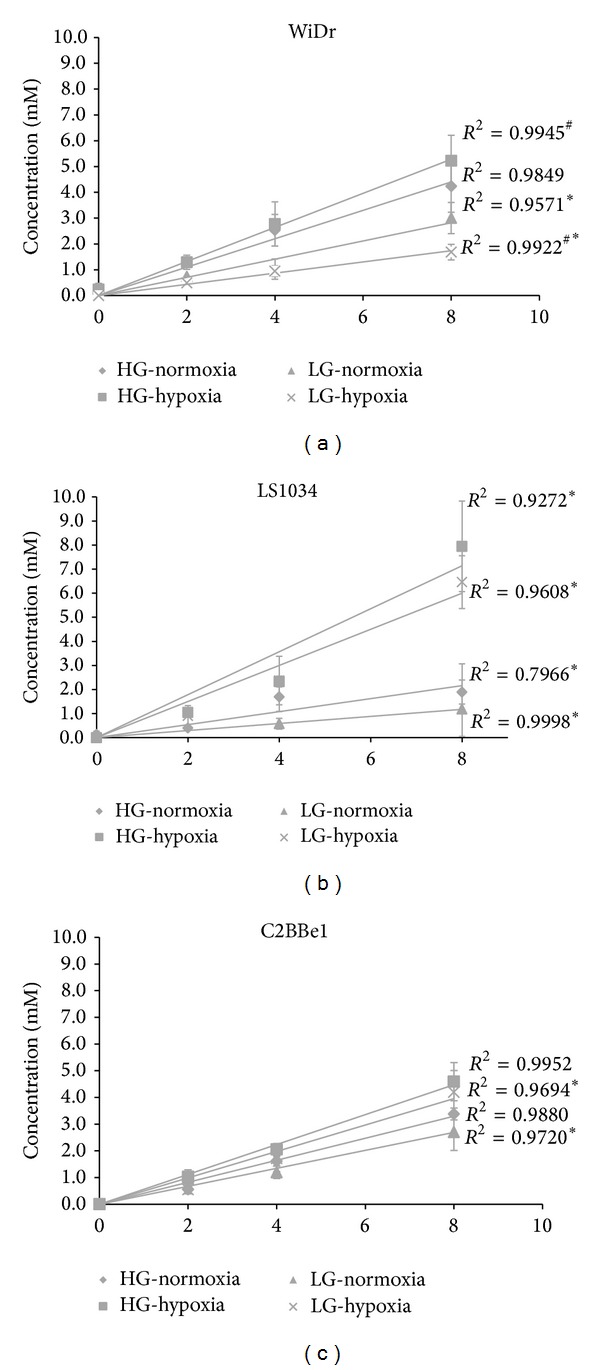
[U-^13^C]lactate exported by colon cancer cell lines to the incubation media during the first 8 hours of incubation under normoxia and hypoxia and low and high glucose media: (a) WiDr; (b) LS1034; (c) C2BBe1. Significant differences are indicated by ∗ corresponding to *P* < 0.05 (comparison between normoxia and hypoxia conditions) and # corresponding to *P* < 0.05 (comparison between high and low cell media glucose concentrations (5 and 25 mM)).

Levels of lactate in cell culture media were monitored by ^1^H-NMR spectroscopy.  Figure 2 shows the temporal evolution of lactate resonances for one of the cell lines (LS1034). This multiplet (duplet of triplets) represents one of the ^13^C-satellites of the methyl resonance of lactate and constitutes half of the signal due to [U-^13^C]lactate.  Figure 3 shows the lactate production for all cell lines under all experimental conditions, high/low glucose and normoxia/hypoxia. A linear evolution is seen for all experimental conditions and for all colorectal cancer cell lines for the first 8 h of incubation. After this period there is a considerable reduction in lactate production which is also consistent with a significant reduction in glucose levels in the culture media. Under normoxia, the three cell lines exhibit a very pronounced glycolytic metabolic profile, exporting considerable amounts of lactate to the culture media. This “Warburg” like metabolic behavior is characteristic of many tumor cells and was expectable for the colorectal cancer cell lines under study. From the three, LS1034 is the less glycolytic followed by C2BBe1 and ultimately WiDr appears as the most glycolytic at both high and low glucose normoxic conditions. This metabolic profile is dramatically altered under hypoxia. This “insult” has completely differential effects in the three cell lines and shows as well a dependence on the glucose content of the cell culture media. The LS1034 cell line is the most susceptible to hypoxia (Figure 3(b)). During the first 8 hours of incubation, lactate production rates increase significantly, more than triplicate, while for other cell lines the increases in those rates are much less notorious. The colorectal cancer cell line with the lowest dependence on glycolysis/lactic fermentation under normoxia becomes the most glycolytic under hypoxia. The amount of glucose in the cell culture media also influences the rates of lactate production by the three cell lines. The combination of normoxia/hypoxia and high/low glucose forms a matrix that seemingly affects differently each of the colorectal cancer cell lines. LS1034 shows a significant increase in lactate production due to hypoxia at both high and low glucose contents while WiDr behaves opposingly for the two glucose levels, increasing glycolysis at high glucose but reducing glycolysis with significant decrease for low glucose, assuming an adaptive behavior. The C2BBe1 is the less affected by the hypoxic insult at both high and low glucose levels, however, with significance at low levels of glucose.

### 3.2. Glycolytic Flux versus Proliferative Activities

**Figure 4:**
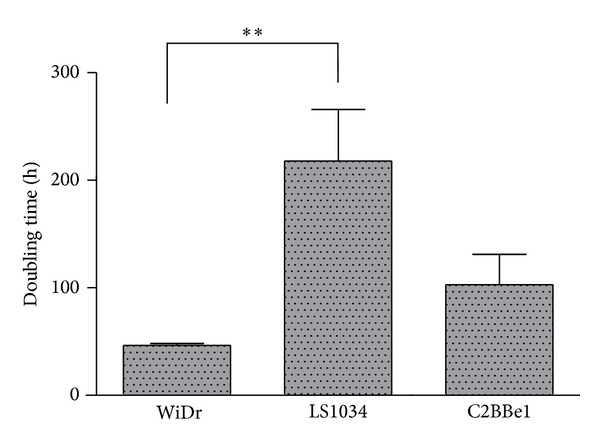
Duplication times (h) for the three colorectal cancer cell lines.

A significant glycolytic activity is expectable in high proliferative tissues for the sake of availability of carbons skeletons for biosynthetic routes. On this basis one would expect that the more proliferative a given cell is, the more glycolytic its metabolic profile should be.  Figure 4 shows the duplication times for the three colorectal cell lines. WiDr is the most proliferative followed by the C2BBe1 and LS1034 appears as the cancer line with the lowest proliferation rate, in agreement with its lowest glycolytic activity as demonstrated by the lactate production rates measured under normoxia with either high or low glucose in culture media. Duplication times and glycolytic activities fully match the concept that there is no “wasteful” metabolic behavior by the cancer cell lines but solely an adaptive behavior that grants them advantage over normal neighboring cells.

### 3.3. Krebs Cycle Kinetics

**Figure 5:**
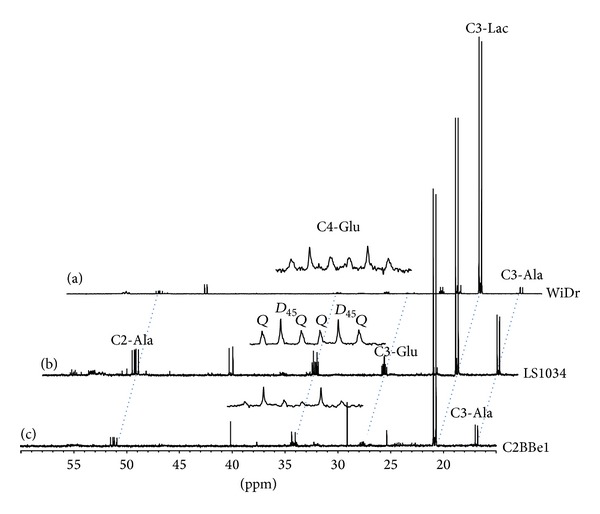
^13^C NMR spectra of perchloric acid extracts for the three colorectal cancer cell lines under normoxia and high glucose media: (a) WiDr; (b) LS1034; (c) C2BBe1. Expanded is the multiplet due to glutamate carbon 4 (C4-Glu; 34.2 ppm), composed by two major multiplets, duplet 45 (*D*_45_), due to glutamate isotopomers labeled in carbons 4 and 5 but not carbon 3, and quartet (*Q*) representing all glutamate isotopomers with those three carbons enriched simultaneously. Other resonances easily seen are the methyl carbons of lactate (C3-Lac; 20.8 ppm) and alanine (C3-Ala; 17.0 ppm) and the carbon 3 of glutamate (C3-Glu; 27.8 ppm).

Krebs cycle kinetics was evaluated by ^13^C-NMR isotopomer analysis using cell perchloric acid extracts.  Figure 5 shows representative ^13^C-NMR spectra of perchloric acid extracts derived from the three colorectal cancer cell lines grown in normoxic conditions in the presence of high glucose. Some of the resonances are identified and the expansions show the multiplet of glutamate C4 resonance (C4-Glu; 34.2 ppm), a crucial carbon for performing Krebs cycle kinetics analysis [14, 16]. Immediately perceptible is the fact that the resonance due to lactate methyl carbon (C3-Lac; 20.8 ppm) dominates all three spectra, corroborating the robust glycolytic/fermentative character of these colorectal cancer cell lines even under normoxia, as outlined above from the rates of lactate production. Other major differences between cell lines are the amounts of alanine (C3-Ala; 17.0 ppm) present in cell extracts, much higher in LS1034, and the composition of the C4-Glu multiplet in terms of duplet 45 (*D*_45_) and quartet (*Q*) multiplets, which as mentioned correlate with Krebs cycle turnover rates. The levels of alanine are intimately associated with the levels of pyruvate in the cells and can be used instead for deriving information concerning the cytosolic redox status [19]. In fact, C3-Lac/C3-Ala is frequently used as an indirect measure of NADH/NAD^+^ ratio [20, 21]. The more the NADH/H^+^ exists in cytosol the more extensive the conversion of pyruvate is to lactate through lactic dehydrogenase (LDH) and the higher the ratio is of those two metabolites. In terms of C4-Glu, the appearance of the* Q* multiplet is only possible after multiple turns of the Krebs cycle (Figure 1) and its contribution to the overall C4-Glu multiplet is higher in cells with a more active oxidative metabolism. The ratio C4*Q*/C4*D*_45_ provides a way to probe Krebs cycle kinetics and to compare different cell lines in terms of their glycolytic/oxidative metabolic characters. Another ratio of metabolic interest is the C3-Lac/C4-Glu, since it reports how enriched the glycolytic/oxidative metabolite pools are and in such a way provides a measure of the metabolic coupling between glycolysis and Krebs cycle. The higher the ratio is the more uncoupled those two central metabolic pathways are and the more dependent the cell line is on glycolysis for its bioenergetics requirements. This ratio is frequently high in “Warburg” like cancer cell lines like the ones used in this study.

**Figure 6:**
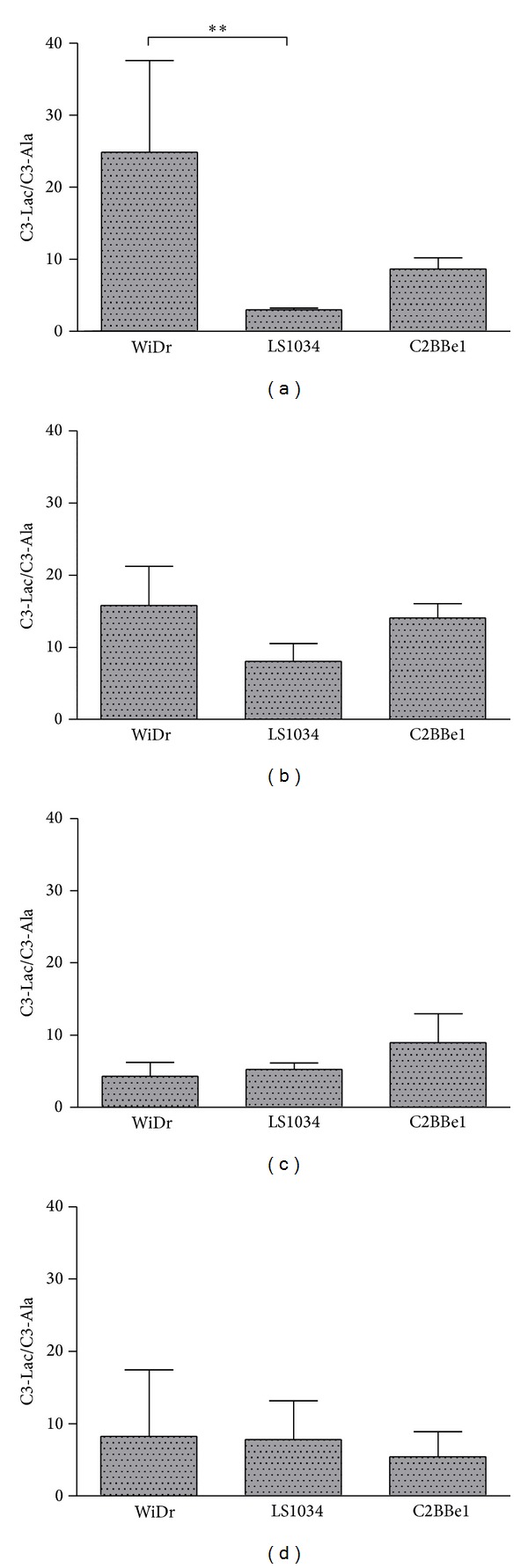
Ratios of the methyl carbon of lactate (C3-Lac) to the methyl carbon of alanine (C3-Ala) for the three colorectal cancer cell lines under all experimental conditions: (a) normoxia/high glucose; (b) hypoxia/high glucose; (c) normoxia/low glucose; (d) hypoxia/low glucose.

Figure 6 shows the ratios of C3-Lac/C3-Ala for the three colon cancer cell lines in the four experimental conditions. This ratio is significantly higher in WiDr under normoxic/high glucose conditions than in the other two cell lines. This correlates with a more pronounced glycolytic flux in this cancer cell line under such conditions. Under hypoxia and high glucose, there is a considerable attenuation of C3-Lac/C3-Ala ratio in WiDr, only explainable by an overall metabolic inhibition in these cells. For LS1034 and C2BBe1 there is an increase in the C3-Lac/C3-Ala ratio under hypoxia, explainable by a higher dependence on glycolysis for bioenergetics under anaerobic conditions.

**Figure 7:**
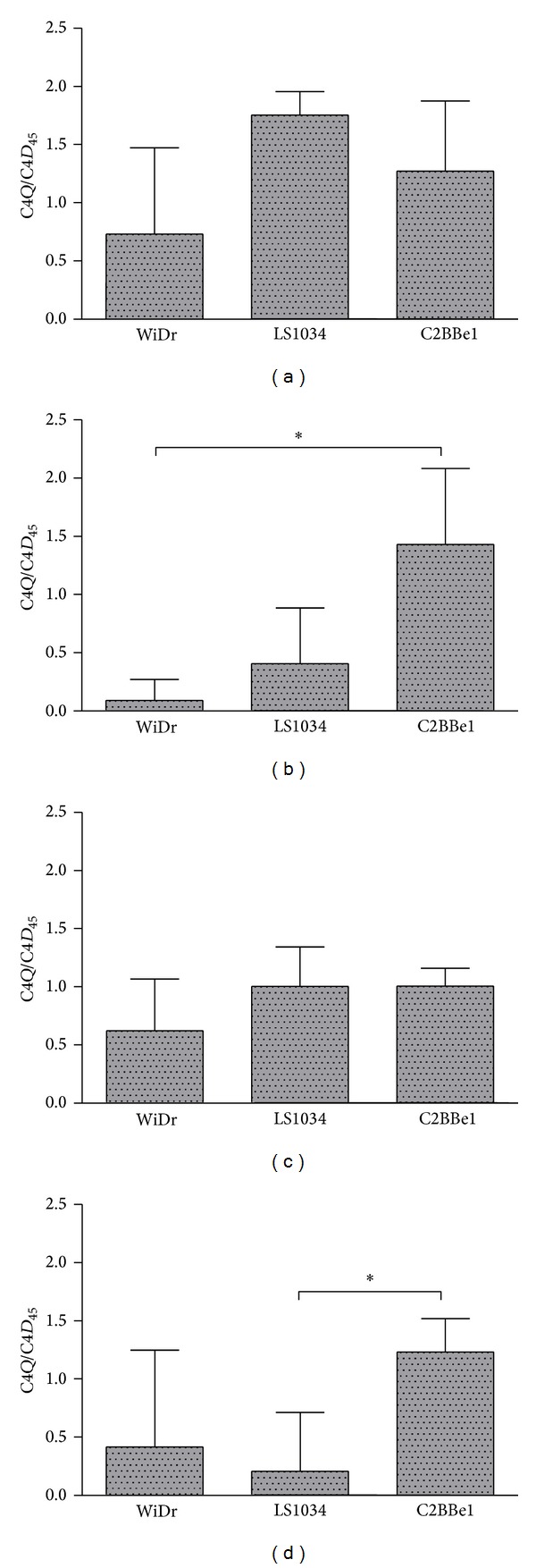
Ratios of the glutamate C4 quartet (C4*Q*) to the duplet 45 (*D*_45_) multiplets for the three colorectal cancer cell lines under all experimental conditions: (a) normoxia/high glucose; (b) hypoxia/high glucose; (c) normoxia/low glucose; (d) hypoxia/low glucose.

Figure 7 shows the ratios C4*Q*/C4*D*_45_ for the three colorectal cancer cell lines in the four experimental conditions chosen. This ratio clearly emphasizes that the three cell lines have distinct Krebs cycle turnovers under hypoxia. WiDr and LS1034 cell lines suffer a considerable reduction in such ratio at both high and low glucose conditions. The same does not hold however for the C2BBe1, which essentially shows no effect in such ratio due to hypoxia. This distinctive behavior emphasizes a distinct capacity of the cells for usage of oxygen at low concentration levels such as the ones prompted by the hypoxic episode and concomitantly justifies the distinctive behavior of cells in tumors when subjected to hypoxia [22].

**Figure 8:**
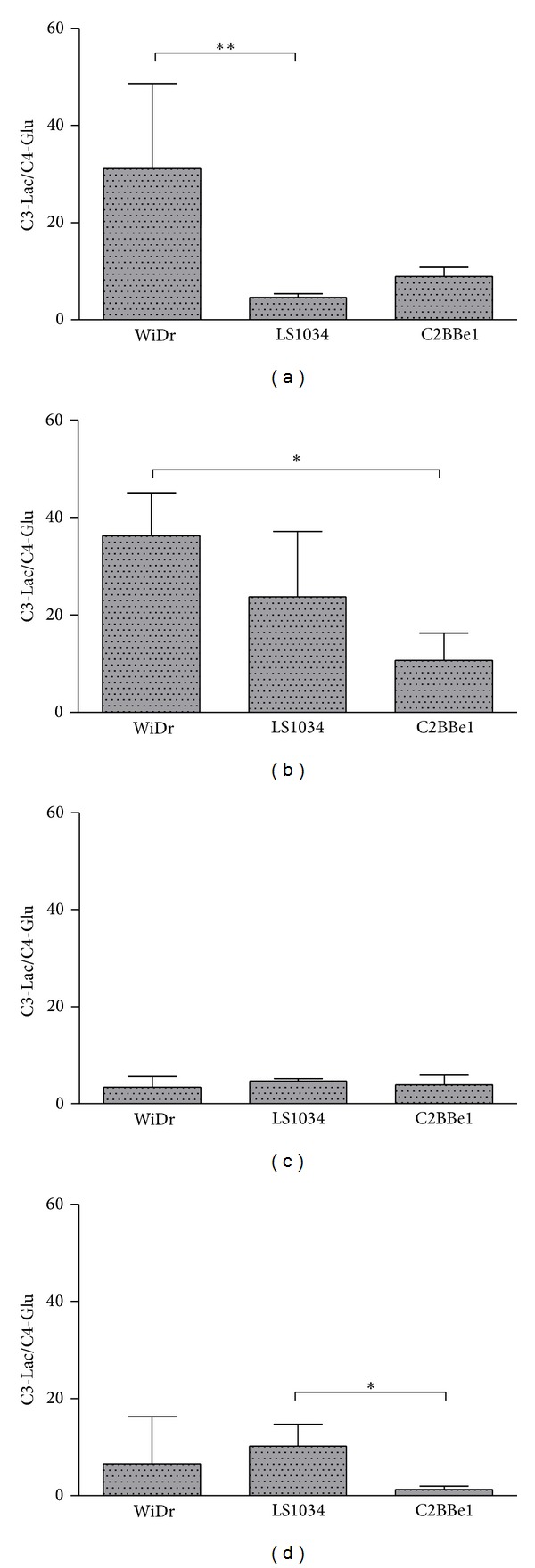
Ratios of the methyl carbon of lactate (C3-Lac) to the carbon 4 of glutamate (C4-Glu) for the three colorectal cancer cell lines under all experimental conditions: (a) normoxia/high glucose; (b) hypoxia/high glucose; (c) normoxia/low glucose; (d) hypoxia/low glucose.

Figure 8 shows the C3-Lac/C4-Glu ratio for the three colon cancer cell lines in the four experimental conditions. As expected, this ratio is higher for more glycolytic cell lines, like WiDr, and suffers a slight increase under hypoxia. Under normoxia and high glucose the LS1034 has the lowest C3-Lac/C4-Glu ratio in accordance with a much more pronounced oxidative profile. Upon hypoxia the LS1034 is the one suffering the more severe metabolic shift since the oxidative pathway reduces its contribution to cell bioenergetics and has to be compensated by further glycolytic activity.

### 3.4. Complex IV Activities

**Figure 9:**
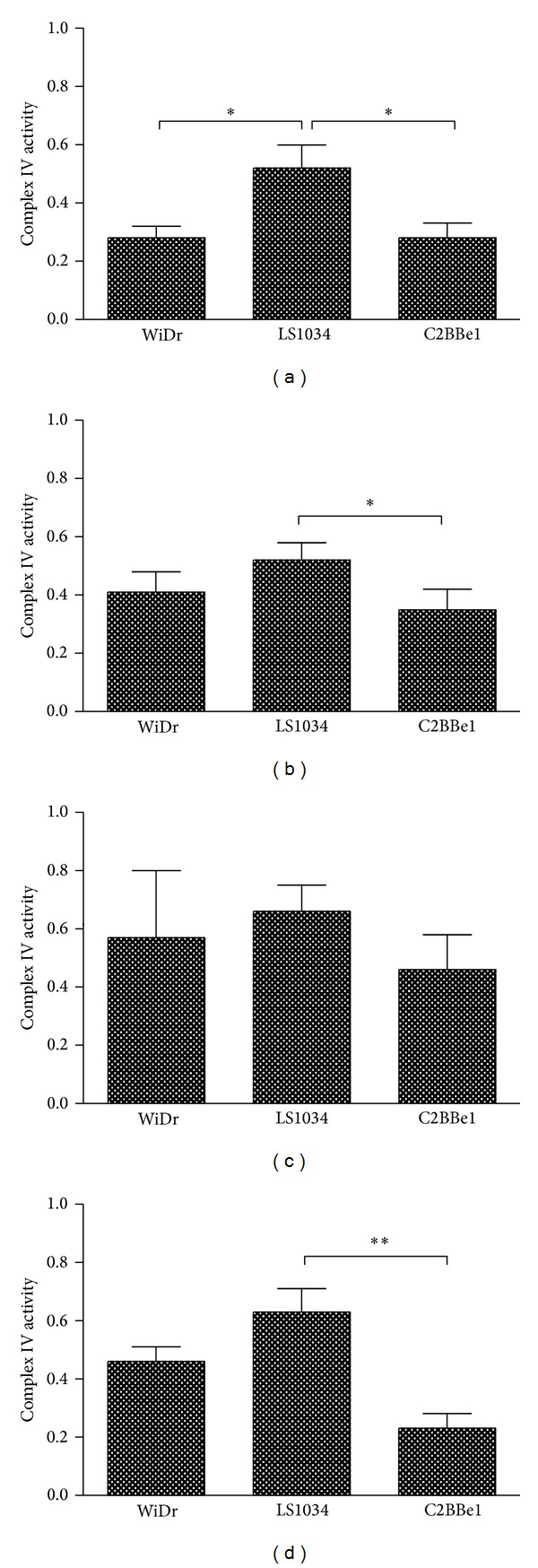
Complex IV activities for the three colorectal cancer cell lines under all experimental conditions: (a) normoxia/high glucose; (b) hypoxia/high glucose; (c) normoxia/low glucose; (d) hypoxia/low glucose.

In order to corroborate the metabolic alterations mentioned above for each of the colorectal cancer cell lines and strengthen our findings concerning the metabolic (un)coupling observed for each of the cell lines in each of the metabolic conditions, the activity of complex IV from the electron transport chain was also evaluated. This activity, adequately normalized for citrate synthase activity, is presented in Figure 9. Under normoxia and high glucose, LS1034 cell line is the one possessing the most active complex IV. This is in accordance with all presented data, which pointed these cells as the most oxidative/less glycolytic under such incubation conditions. Under normoxia and low glucose no significant changes were detected in complex IV activities in accordance with the absence of significant differences on the ratios C3-Ala/C4-Glu and C4*Q*/C4*D*_45_ discussed above for this experimental condition. Low substrate concentration does not seem by itself to be able to distinguish the three colorectal cell lines under analysis. Hypoxia on the other hand prompts for distinction even at low glucose levels, thus emphasizing the distinctive metabolic character of each colorectal cancer cell line. This distinct responsiveness is a crucial parameter to be evaluated aiming at the implementation of more efficient anticancer therapeutic strategies.

The implemented methodology for analysis of both culture media and perchloric acid cellular extracts provides unique and complimentary metabolic information, which affords a more complete picture of the metabolic transformations associated with each of the specific actions undertaken for challenging cancer cell metabolism. This methodology can naturally be extended to other metabolic pathways and metabolic tracers towards widening the scopes of the current metabolic analysis.

## 4. Conclusions 

The three colorectal cancer cell lines show very distinct metabolic profiles in the four experimental conditions tested. Glycolysis is in every circumstance the most active metabolic pathway denoting the “Warburg” like metabolic behavior that characterizes many cancer cells. Nevertheless, oxidative flux through the Krebs cycle assumes its importance in whole cellular bioenergetics and its extent is itself very distinct among the three colorectal cancer cell lines. The interplay of oxygen (normoxia versus hypoxia) and nutrient (high versus low glucose) availability also plays a crucial role in the definition of cell capacity to adapt to distinct microenvironments. In this study the (in)sensibility to the three colorectal cancer cell lines was surprisingly different. The less glycolytic under normoxia, LS1034, turns into the most glycolytic in hypoxia while the most glycolytic barely respond to the hypoxic insult. Also, the alteration in nutrient availability interferes in the metabolic coupling between glycolysis and Krebs cycle and demonstrates the distinct capacity that cells have to react to such interferences. We understand that the whole picture is much more complex but would like to point out that the simple management of O_2_ and glucose constitutes certainly a mechanism to take into account to tackle tumor proliferation and devise more efficient therapeutic strategies. The adequate knowledge of the metabolic behavior of cancer cells is thus a must on this “crusade” against cancer and the methodology here described is certainly pivotal for deriving this much-needed information.
